# Whole exome sequencing identifies a novel intron heterozygous mutation in *TSC2* responsible for tuberous sclerosis complex

**DOI:** 10.1038/s41598-019-38898-9

**Published:** 2019-03-14

**Authors:** Yicong Ye, Yong Zeng

**Affiliations:** 1 Department of Cardiology, Beijing Institute of Heart, Lung and Blood Vessel Diseases, Beijing Anzhen Hospital, Capital Medical University, Beijing, 100029 China; 2 Department of Cardiology, Chinese Academy of Medical College and Peking Union Medical College Hospital; Peking Union Medical College Hospital, Beijing, 100730 China

## Abstract

This study was aimed to identify the potentially pathogenic gene variants that contribute to the etiology of the tuberous sclerosis complex. A Chinese pedigree with tuberous sclerosis complex was collected and the exomes of two affected individuals were sequenced using the whole exome sequencing technology. The resulting variants from whole exome sequencing were filtered by basic and advanced biological information analysis and the candidate mutation was verified as heterozygous by sanger sequencing. After basic and advanced biological information analysis, a total of 9 single nucleotide variants were identified, which were all follow the dominant inheritance pattern. Among which, the intron heterozygous mutation c.600-145 C > T transition in *TSC2* was identified and validated in the two affected individuals. *In silico* analysis with human splicing finder (HSF) predicted the effect of the c.600-145 C > T mutations on *TSC2* mRNA splicing, and detected the creation of a new exonic cryptic donor site, which would result in a frame-shift, and finally premature termination codon. Our results reported the novel intron heterozygous mutation c.600-145 C > T in *TSC2* may contribute to TSC, expanding our understanding of the causally relevant genes for this disorder.

## Introduction

Tuberous sclerosis complex (TSC) is an autosomal dominant (95% penetrance) neurocutaneous and progressive disorder, commonly characterized by the occurrence of various tumors in different organs^1^. It is reported that two-thirds of TSC cases are sporadic, which reflects a high spontaneous mutation rate^2^. TSC can affect people of all age groups with multiple organ systems involved in different ways and at varying time^3^. The clinical presentation of TSC varies greatly even within a given family^4–6^.

Multisystem hamartomatous lesions in the brain, skin, kidney, lung, retina and heart are very common. The central nervous system is the most severely and commonly affected organ system in TSC patients. Cortical tubers, subependymal nodules and subependymal giant cell astrocytomas are the main structural brain lesions^4,5^. It is pointed out that tubers growing in the brain are closely associated with high morbidity and mortality of TSC^7^. Skin lesions are detected in most of TSC patients and include shagreen patches, hypomelanotic macules, confetti-like lesions, facial angiofibromas, forehead fibrous plaque and periungual and ungual fibromas^8^. After central nervous system and skin findings, renal manifestation is the most common abnormality in TSC patients^9^. Pulmonary involvement, especially lymphangioleiomyomatosis, is the third most common cause of TSC-associated morbidity^9^. TSC is also related to both retinal and nonretinal ocular findings^10^. Moreover, hamartomas are the most common retinal manifestation of TSC^9^. In addition, various cardiac rhabdomyomas are occurred in TSC patients^9^. The disease severity of TSC is variable with signs and symptoms ranging from hypomelanotic macules, to epilepsy, autism, intellectual disability and multiple hamartomas in brain, kidney, lung and heart^11^.

The phenotypic expression of TSC is highly variable and sometimes it can be difficult to establish the definitive clinical diagnosis. Recently, mutation analysis has become an additional diagnostic tool in TSC. It has been demonstrated that TSC is caused by mutations in either the *TSC1* gene on chromosome 9q34, or the *TSC2* gene on chromosome 16p13.3^12,13^. It is worth mentioning that several *TSC2* variants including A1801G, F143L, S132C, A196T, Y598H, C244R, T993M, L1511H and R1772C have been identified in individuals with symptoms of TSC^14,15^. Considering the genetic heterogeneity, the identity of the novel candidate genes remains a challenge. In the current study, we used whole exome sequencing to identify the novel causative gene for the two affected individuals in a Chinese TSC family. Our study may improve the understanding of this disorder and provide insight into the genetic basis for inherited TSC.

## Materials and Methods

### Human subjects

For the purpose of this study, a four-generation Chinese tuberous sclerosis (TSC) family with five affected individuals and five unaffected individuals was recruited. Given high suspicion for the TSC family, the two affected individuals and one unaffected individual were enrolled for the exome sequencing screen. The blood samples were collected from the participants for DNA extraction. All experiments were performed in accordance with relevant guidelines and regulations. The written informed consent was obtained from study subjects or guardian before the study. The study was approved by the licensing committee of Beijing Anzhen Hospital.

### Analysis of exome capture

The genomic DNA was extracted from the blood samples according to the standard procedures. The 2 μg of genomic DNA was fragmented with about 200 bp, then ligated with adapters and amplified by ligation-mediated PCR. The qualified genomic DNA was used for exome capture and high-throughput sequencing. Agilent SureSelect Human All Exon 50 Mb Exon Kit was used to perform exome target enrichment. The captured library was sequenced on the Illumina Hiseq4000 sequencer with paired-end 125-bp and mean coverage of 100×.

### Analysis of basic biological information

The fastQC was used to evaluate the quality of raw sequencing data of exome sequencing. Under tools of SeqPrep and sickle, raw data was filtered by removing adapter, contaminating reads and low quality reads, and remains were the clean ones. The exome sequencing clean reads were mapped to the reference human genome sequence (hg19) (http://hgdownload.soe.ucsc.edu/goldenPath/hg19/bigZips/) using the Burrows-Wheeler Alignment (BWA) tool (http://bio-bwa.sourceforge.net/bwa.shtml), which can do short reads alignment to a reference genome and support paired-end mapping. The sequence alignment/map (SAM) file was then generated. Picard tool (http://picard.sourceforge.net/) was used to mark and exclude the duplicate reads. Variants (single nucleotide variants (SNVs), insertions and deletions) calling was performed using the Genome Analysis Toolkit (GATK)^16^.

### Analysis of advanced biological information

In this process, we performed the analysis of dominance/recessiveness screening and mutation site screening. To find the potential important variants, the ANNOVAR tool (http://www.openbioinformatics.org/annovar/) was used to annotate the resulting SNVs^17^, and the information for variant frequencies and location within genes were obtained. Moreover, the SNVs were sequentially filtered and given higher priority with the following criteria: (1) Quality By Depth (QD) < 2.0, Phred-scaled p-value using Fisher’s exact test to detect strand bias (FS) > 60.0, Mapping quality (MQ) < 40.0, Z-score From Wilcoxon rank sum test of Alt vs. Ref read mapping qualities (MQRankSum) < −12.5 and Z-score from Wilcoxon rank sum test of Alt vs. Ref read position bias (ReadPosRankSum) < −8.0; (2) minor allele frequency (MAF) < 0.05 in 1000 Genomes Project; (3) damaging as predicted by 6 bioinformatics programs including PhyloP (score > 0.85), SIFT (score < 0.05), PolyPhen (score > 0.85), GERP (score > 2), Mutation Taster (score > 0.5), and LRT (score > 0.9); (4) consistent with model of dominant disease transmission.

### Variant validation

To validate the variants identified through exome sequencing, candidate SNVs were selected for sanger sequencing. The blood samples were obtained from the selected individuals. Genomic DNA was extracted and SNVs were tested in the original three individuals who underwent exome sequencing and three additional unaffected individuals in the four-generation Chinese TSC family.

### Splicing analysis of variant

Human Splicing Finder (HSF) (http://www.umd.be/HSF/) is a tool to predict the effects of mutations on splicing signals, which could identify splicing motifs and evaluate the strength of branch points in any human sequence. In this study, we used this tool to predict the effects of identified mutations on mRNA splicing based on the method in the previous report of HSF use^18^. The detailed process of our analysis is as follows:

In order to analyze for the presence and predicted strengths of splice sites, we first chose the analysis type as “Splice site analysis” along with the option of “Automatically select the longest transcript”, and then pasted base sequence (50 bp upstream and downstream of the wild-type or variant *TSC2* genomic DNA sequence) into the analysis box. Lastly, we chose the mutation position as “64” and type of mutation as “substitution”. In the end, we got the result of “Sequences” and “Interpreted data”. From the “Sequences”, we got the reference sequence and mutant sequence. From the “Interpreted data”, we found the results of the predicted signal, prediction algorithm, cDNA position and interpretation.

## Results

### Information of proband

**Figure 1:**
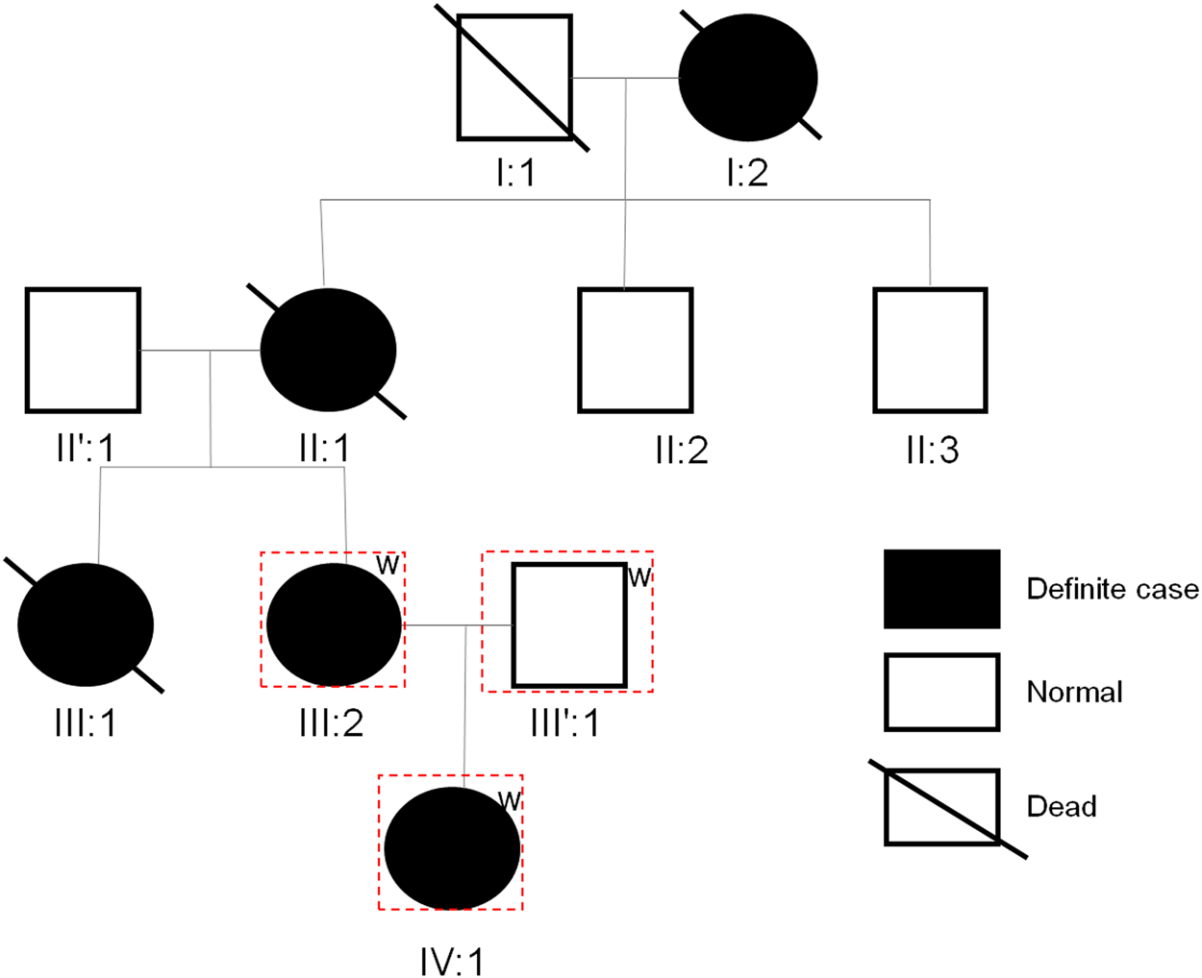
Pedigree for the Chinese family with TSC. Individuals III:2, III′:1 and IV:1 underwent exome sequencing. W: exome sequencing.

**Figure 2:**
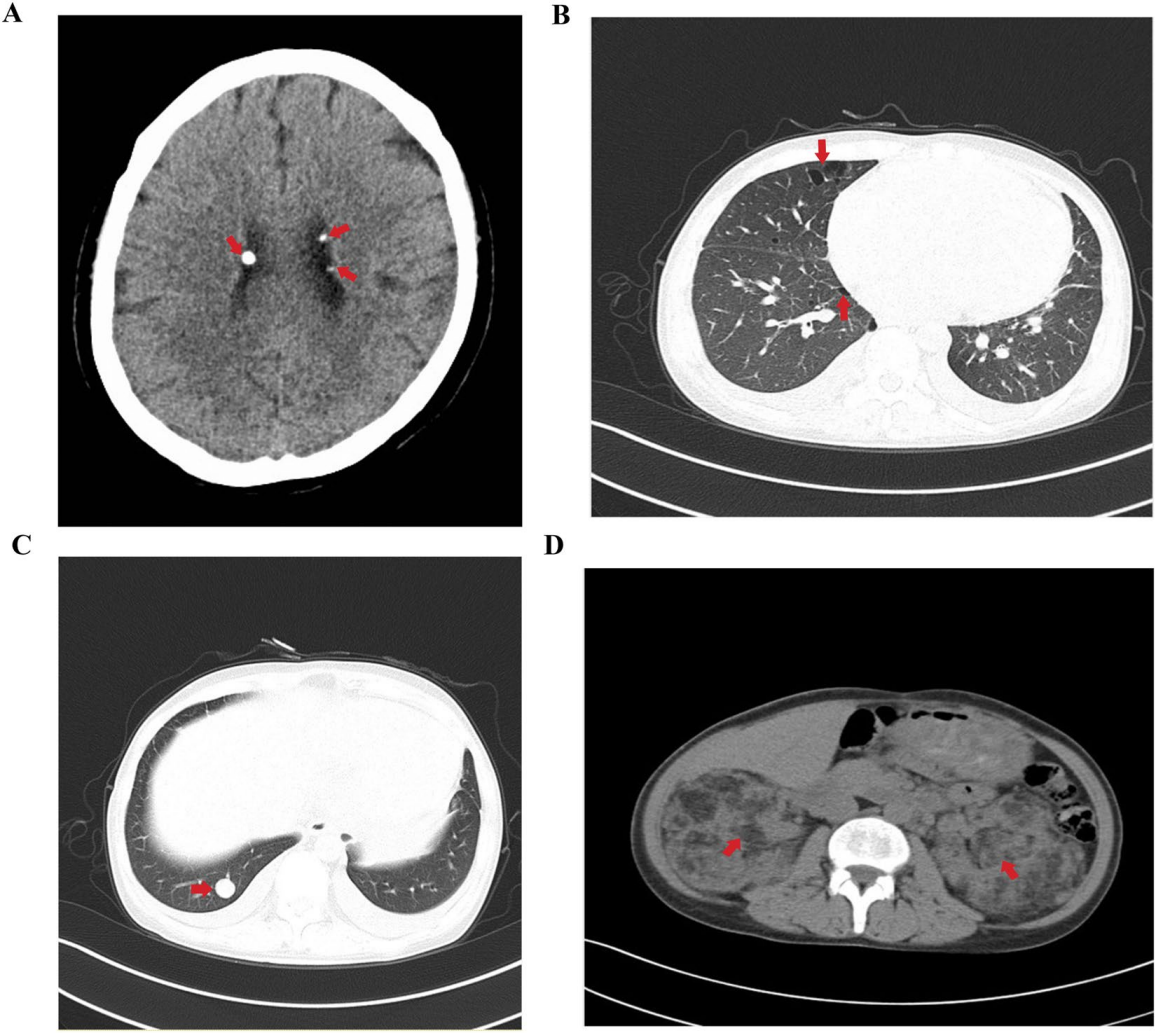
The CT scan of the proband’s head, lung, and abdomen. (**A**) The head CT scan of the proband. Nodular calcifications in the bilateral ventricle (arrow). (**B**) The lung HRCT scan of the proband. Multiple bullae in right lung (arrow). (**C**) The lung HRCT scan of the proband. Pulmonary nodule in right lung (arrow). (**D**) The abdominal CT scan of the proband. Bilateral renal angiomyolipoma (arrow).

We studied a Chinese family affected with TSC, in which there were five affected individuals (Sample I:2, II:1, III:1, III:2 and IV:1) (Fig. 1). The proband (III:2) was a thirty-nine-year-old woman who presented with TSC. Moreover, her mother (II:1) and grandmother (I:2) were also presented with TSC with similar phenotypes. In addition, her little sister (III:1) had been dead of epilepsy. However, the grandfather (I:1), father (II′:1), two uncles (II:2 and II:3) and spouse (III′:1) of the proband were asymptomatic. The proband developed from childhood and accompanied with coronary heart disease and polycystic kidney disease. The skin of the proband showed coffee and milk stains. The head CT scan of the proband showed low density in left caudate nucleus and right frontal cortex, multiple nodular and nodular calcifications in the left caudate nucleus head, anterior border of left cerebellar hemisphere, left temporal lobe and bilateral ventricle (Fig. 2A), multiple nodules and patchy high-density shadows in the right temprral lobe and the left frontal cortex, and microchip low density on the left side of the parietal bone. The score of mini-mental state examination was 25.

The lung HRCT scan of the proband showed multiple bullae and pulmonary nodules in right lung (Fig. 2B,C), enlarged axillary and mediastinal lymph nodes, increased heart size, pericardial effusion, and bilateral pleural effusion. The pulmonary first pass imaging indicated that no signs of pulmonary hypertension and right-left shunt were seen.

The abdominal CT scan of the proband showed that bilateral masses with multiple hypodense (angiomyolipoma) were identified in bilateral kidney area instead of normal kidneys (Fig. 2D).

### Identification of candidate genes

According to the TSC pedigree, we speculated that TSC was dominant inheritance. The pathogenic gene in the proband may be from her mother and grandmother. Therefore, exome sequencing was ideally suited to screen for the causal genes of the TSC pedigree. The whole exomes of III:2, III′:1 and IV:1 were sequenced, followed by variant detection and filtering. The exome sequencing led to the detection of 47687, 48539 and 48795 SNVs for III′:1, III:2 and IV:1 (Table 1). After further analysis of dominance/recessiveness screening and mutation site screening, a total of 9 SNVs were identified, which were all follow the dominant inheritance pattern. Detailed information of 9 SNVs was showed in Table 2. Among which, *TSC2* is an intron heterozygous mutation gene, which was a rare event in the TSC. Therefore, we focused on *TSC2* gene in this study.

**Table 1:** The number of SNVs in different regions of genome after exome sequencing.

	Exonic	Intronic	UTR3	UTR5	Splicing	Intergenic	NcRNA_ exonic	NcRNA_ intronic	NcRNA_ splicing	NcRNA _UTR3	NcRNA _UTR5	Up stream	Down stream	total
III′:1	20030	16757	3857	1821	56	2065	1181	797	3	104	51	605	360	47687
III:2	20008	16977	4274	1846	60	2165	1179	861	4	131	38	613	383	48539
IV:1	19959	16994	4448	1846	50	2192	1225	849	5	149	46	636	396	48795

Exonic: exon region; Intronic: intron region; UTR3: 3′UTR region; UTR5: 5′UTR region; Splicing: splicing junction 10 bp region; Intergenic: intergenic region; NcRNA exonic: non-coding RNA exon region; NcRNA intronic: non-coding RNA intron region; NcRNA intronic: non-coding RNA splicing junction 10 bp region; Up stream: the upstream 1 Kb region of the transcription initiation site; Down stream: the downstream 1 Kb region of the transcription initiation site.

**Table 2:** Finally identified 9 SNVs in exome sequencing.

Gene	Detail_information	Chromosome	Start	End	Ref	Alt	DbSNP138
C2orf82	C2orf82:NM_206895:exon1:c.C19G:p.L7V	Chr2	233735070	233735070	C	G	rs200597442
RPGRIP1	RPGRIP1:NM_020366:exon10:c.C1295T:p.S432F	Chr14	21785998	21785998	C	T	rs190985984
FAM160B2	FAM160B2:NM_022749:exon9:c.G1084T:p.D362Y	Chr8	21956804	21956804	G	T	rs199982834
IGSF3	IGSF3:NM_001007237:exon9:c.G2836A:p.V946I,IGSF3:NM_001542:exon9:c.G2896A:p.V966I	Chr1	117127279	117127279	C	T	rs192954398
DTHD1	DTHD1:NM_001136536:exon2:c.T56G:p.V19G,DTHD1:NM_001170700:exon2:c.T551G:p.V184G	Chr4	36292033	36292033	T	G	rs77539527
PDZD3	PDZD3:NM_001168468:exon7:c.C721T:p.R241C,PDZD3:NM_024791:exon7:c.C679T:p.R227C	Chr11	119058712	119058712	C	T	rs147651078
LAMTOR4	LAMTOR4:NM_001008395:exon4:c.G249T:p.R83S	Chr7	99751536	99751536	G	T	NA
RNF152	RNF152:NM_173557:exon2:c.C494T:p.T165I	Chr18	59483203	59483203	G	A	NA
TSC2	TSC2:NM_001318194:intron:c.C > T	Chr16	2106052	2106052	C	T	NA

Chr: chromosome; Ref: reference allele; Alt: alteration allele; rs: accession number in dbSNP138; NA: not applicable.

### Sanger sequencing of *TSC2* variants

**Figure 3:**
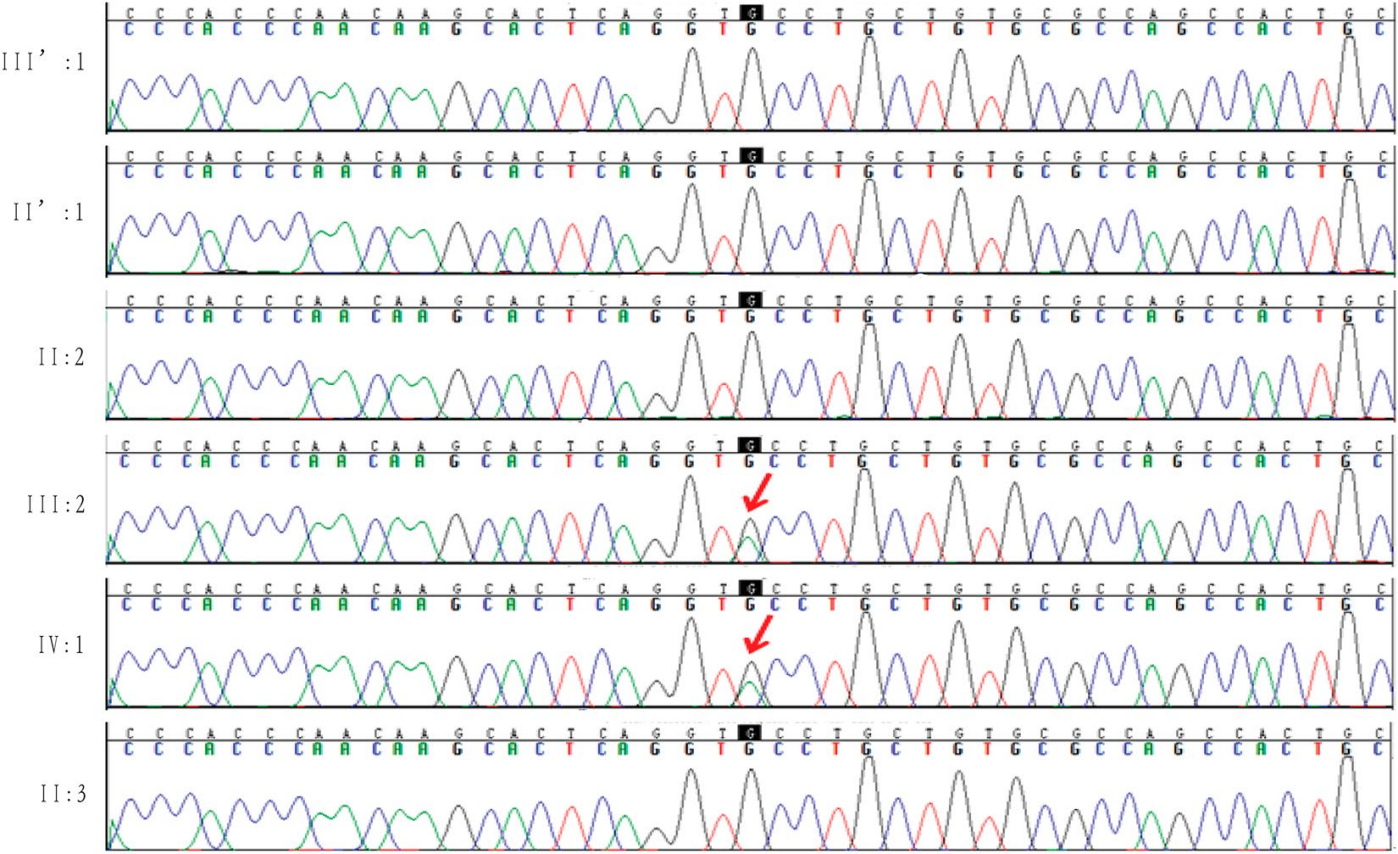
Sanger validation results of TSC2 variants. Red arrow presented the mutation site.

To further confirm the variant of c.600-145 C > T in *TSC2* in TSC, sanger sequencing was performed in the original three individuals (III:2, III′:1 and IV:1) who underwent exome sequencing and three unaffected individuals (II′:1, II:2 and II:3) in the TSC family. The results showed that the variant was confirmed as heterozygous in the affected proband (III:2) and her daughter (IV:1) and as wild type in four unaffected individuals (III′:1, II′:1, II:2 and II:3) via Sanger sequencing (Fig. 3), which further demonstrated that the variant of c.600-145 C > T in *TSC2* was closely associated with TSC.

### *In silico* analysis of *TSC2* variant

*In silico* analysis with a freely available online bioinformatics tool, human splicing finder (HSF) (http://www.umd.be/HSF3/) predicted the effect of the c.600-145 C > T mutations on *TSC2* mRNA splicing. The HSF analysis detected the creation of a new exonic cryptic donor site, generating consensus values of 52.35 and 79.18 for the wild-type and mutant c.600-145 C > T nucleotides, respectively. The predicted consensus value deviation of +51.25% for the new exonic cryptic donor site indicates the loss of the wild-type splice site which would result in a frame-shift, and finally lead to a premature stop codon in the protein.

## Discussion

The phenotypic expression of TSC is highly variable and sometimes it is difficult to establish a definitive clinical diagnosis. It is noted that mutation analysis has become an important diagnostic tool in familial as well as sporadic TSC. In this study, whole-exome sequencing was performed on the two affected individuals in a Chinese TSC pedigree, identifying a novel intron heterozygous mutation in *TSC2* (c.600-145 C > T). Our result further demonstrated the crucial role of *TSC2* in the development of TSC. The *TSC2* gene comprises approximately 43 kb of genomic DNA with 41 exons encoding a 5.5 kb transcript and the 198 kDa protein of tuberin. There are various possible mechanisms for somatic inactivation of the wild-type allele of *TSC2*, including mutation, loss of heterozygosity and promoter methylation. It is reported that loss of function mutation in *TSC2* leads to abnormal production of the end products, and finally promotes tumorigenesis of TSC^9^. Dabora S.L. *et al*. found that the disease was usually milder in patients with the TSC phenotype and no identifiable mutation in *TSC2*^2^. In addition, only the p. R905Q mutation in *TSC2* has been found related to milder TSC^9^.

TSC is an autosomal dominant neurocutaneous syndrome caused by mutations of TSC1 or TSC2 genes. Tyburczy M.E. *et al*. reported that 45 of 53 subjects found mutations, and TSC2 mutations and TSC1 mutations account for 82% and 18%, respectively^19^. TSC2 is a common intron heterozygous mutation gene in TSC. In most studies of the identified mutations in the *TSC2* gene are either missense mutations or small and non-truncating insertions/deletions mutations. Heterozygous missense variant c.899 G > T, p.G300V in the *TSC2* gene is found in patients with variable TSC-associated symptoms and signs^20^. The missense variant c.3599 G > C, p.R1200P in *TSC2* gene is identified in the DNA of peripheral leukocytes of TSC patients^21^. It is noted that some missense changes in *TSC2* are related to TSC in definite TSC patients, TSC in familial TSC patients and TSC in which patients symptoms are less severe^22–28^. In addition, the novel deletion mutant c.700–701 in the *TSC2* gene was detected in patients with TSC^29^. In the aspect of signaling pathway, the TSC2 protein functions as a heterodimer to suppress the target of rapamycin mTOR, a serine/threonine protein kinase that play roles in the regulation of cell growth and division^30,31^. It is demonstrated that the small deletion mutation in *TSC2* is associated with severe TSC that promotes mTOR signaling pathway^29^.

Herein, we identified a new intron heterozygous mutation in *TSC2* (c.600-145 C > T) in a Chinese TSC pedigree, which was not reported before. The mutation type will lead to a novel variable splicing site, which might be associated with abnormal function of TSC2 protein.

Alternative splicing is a biological process of post-transcriptional RNA processing whereby the single gene can encode various distinct transcripts, which increases the diversity of mRNAs expression^32,33^. It is showed that alternative splicing can regulate binding between proteins, between proteins and membranes and between proteins and nucleic acids^33^. It is reported that the aberrant regulation of alternative splicing leads to human disease^34–42^. In addition, alternative splicing also plays roles in brain development and is involved in several neurological diseases^43^. Torrado *et al*.^44^ reported a novel intronic mutation (the c.2678-15 C > A variant), within intron 22 of the *FBN1* gene, in a Marfan syndrome (MFS) patient with aortic dilatation. The c.2678-15 C > A variant disrupts normal splicing of intron 22 leading to frameshift, premature termination codon, and finally haploinsufciency of the FBN1 functional protein. In Lynch syndrome families^45,46^, c.[2635-3 T > C;2635-5 C > T] MSH2 mutation, located in intron 15, caused a significant reduction of MSH2 mRNA expression via altering the correct mRNA processing, suggesting a pathogenic role for the variant. Cariola F. *et al*.^46^ also described the variant c.2635-2 A > G in intron 15 of the *MSH2* in with three members of a family manifesting the Lynch syndrome, which affects the splice site consensus sequence, and result in the absence of MSH2/MSH6 heterodimer protein. Yu *et al*. reported that the variant c.772 + 27 G > C in intron 6 of *ACVRL1*gene in a Chinese family with hereditary hemorrhagic telangiectasia (HHT) presents a decreased expression of *ACVRL1* mRNA and protein in affected HHT2 patients^47^. Therefore, we speculated that the intron heterozygous mutation in TSC2 (c.600-145 C > T) may affect the expression of the TSC2-encoded protein tuberin through alternative splicing.

It is indicated that patients with *TSC2* mutations tend to have an earlier onset, more severe cognitive deficits and higher frequency of seizures^46^. Northrup H. *et al*. found that the clearly inactivating *TSC2* mutation was considered as sufficient evidence for TSC diagnosis, even in the absence of clinical signs^47^. Therefore, mutation analysis of the *TSC2* genes in both suspected and definite TSC patients is important in genetic counselling. Our result may be helpful in the diagnosis and genetic counseling of TSC.

In summary, TSC is a complex disease with significant genetic heterogeneity. We demonstrated the presence of a novel intron heterozygous mutation c.600-145 C > T in *TSC2* in the affected individuals, which may potentially contribute to TSC susceptibility. However, there is a limitation of our study. We didn’t perform the pathogenic mechanism study of identified mutation in *TSC2*. The animal model or cell culture experiments are needed to further investigate the potential biological function of *TSC2*.
